# Estimating Vaccine Efficacy Against Transmission via Effect on Viral Load

**DOI:** 10.1097/EDE.0000000000001415

**Published:** 2021-08-30

**Authors:** Lee Kennedy-Shaffer, Rebecca Kahn, Marc Lipsitch

**Affiliations:** From the a Department of Mathematics and Statistics, Vassar College, Poughkeepsie, NY; b Center for Communicable Disease Dynamics, Harvard T H Chan School of Public Health, Boston, MA; c Department of Epidemiology, Harvard T H Chan School of Public Health, Boston, MA.

**Keywords:** Coronavirus disease 2019, SARS-CoV-2, Study design, Transmission, Vaccine efficacy

## Abstract

Determining policies to end the SARS-CoV-2 pandemic will require an understanding of the efficacy and effectiveness (hereafter, efficacy) of vaccines. Beyond the efficacy against severe disease and symptomatic and asymptomatic infection, understanding vaccine efficacy against virus transmission, including efficacy against transmission of different viral variants, will help model epidemic trajectory and determine appropriate control measures. Recent studies have proposed using random virologic testing in individual randomized controlled trials to improve estimation of vaccine efficacy against infection. We propose to further use the viral load measures from these tests to estimate efficacy against transmission. This estimation requires a model of the relationship between viral load and transmissibility and assumptions about the vaccine effect on transmission and the progress of the epidemic. We describe these key assumptions, potential violations of them, and solutions that can be implemented to mitigate these violations. Assessing these assumptions and implementing this random sampling, with viral load measures, will enable better estimation of the crucial measure of vaccine efficacy against transmission.

To understand how vaccinations will affect the severe acute respiratory syndrome coronavirus 2 (SARS-CoV-2) pandemic, we must be able to assess both the direct protection and indirect protection that these vaccines confer.^1^ Individually randomized controlled trials (RCTs) have shown high-vaccine efficacy of several vaccine candidates in preventing symptomatic, laboratory-confirmed disease.^2–5^ Several studies have also demonstrated that the vaccines further protect against asymptomatic infection, as measured through virologic or serologic testing of trial participants^3–5^ and through repeated sampling in longitudinal cohorts.^6^ However, in the absence of perfect efficacy against acquisition of the infection, fully assessing the effect of vaccination requires an assessment of the vaccine’s efficacy at preventing transmission of the pathogen from infected vaccinated individuals to susceptible individuals.^7^ This is especially important for variants, some of which show increased transmissibility,^8^ and in the presence of potential reduced vaccine efficacy against disease for some variants^8,9^ or waning effectiveness with time.^10^

So far, this has been assessed primarily through retrospective observational studies, which have found a reduced risk of infection among household contacts of vaccinated individuals compared with household contacts of unvaccinated individuals.^11,12^ Throughout this article, we discuss estimands that could be estimated in studies that are randomized or observational, “idealized” or “real world.” For brevity, we use the term efficacy throughout, to avoid the various distinctions often made between efficacy and effectiveness.^13–15^

Halloran et al. defined vaccine efficacy on infectiousness and its role in understanding the total and overall vaccine effects.^7^ However, methods to estimate this parameter generally focus on large-scale observational studies with observation of contacts of infected individuals,_13_ add-on household studies, genetic linkage studies, or cluster randomized trials.^1^ Rinta-Kokko et al. described methods to estimate vaccine efficacy on carriage prevalence and infer the efficacy on incidence through measured odds ratios.^16^ More recently, similar methods have been proposed to estimate the vaccine efficacy on incident infection, accounting for vaccine effects that change the duration of infection (and thus, likely, the duration of infectiousness) by incorporating random sampling and testing among RCT participants.^17,18^ Follmann and Fay moreover describe how, by measuring viral loads or a proxy thereof, the vaccine efficacy on transmission could be estimated.^18^

We describe a method, similar to that laid out in Follmann and Fay,^18^ to estimate vaccine efficacy against transmission by estimating both vaccine efficacy against detectable infection and vaccine efficacy against per-contact infectiousness. This process relies on sampling and virologic testing of the full study population or a random sample thereof within a RCT of a vaccine candidate, as was done in at least one vaccine study for COVID-19.^3^ Additionally, observational studies have used differences in viral load, as measured by the cycle threshold (Ct) value of reverse transcriptase-quantitative polymerase chain reaction (RT-qPCR) tests, to suggest that vaccines may reduce the likelihood of transmission,^9,19^ although perhaps not for the B.1.617.2 (Delta) variant.^8,20^ Although Follmann and Fay focus on the inferential method and statistical properties of such an estimator,^18^ we focus here on assumptions about the infection and transmission process and the vaccine effect that are necessary for consistent estimation of this effect and for proper interpretation of observed differences (or lack thereof) in viral load.

In the next section, we describe the formulation of the estimand of interest and its relation to other common vaccine efficacy measures. In the following section, we define an estimator of this effect and show that it is consistent for that estimand. In the “Assumptions for Consistency” section, we detail the assumptions needed for that consistency, describing their implications, examples of possible violations, and approaches that may be used to mitigate these violations. Finally, we describe the implications of this method and the research needed to best understand when these assumptions are met for SARS-CoV-2 and other infectious pathogens.

## ESTIMAND OF INTEREST

We are interested in the vaccine efficacy in preventing transmission, *VE*_*T*_, the reduction in the probability of transmission from an individual at any given time caused by vaccination of that individual. This probability, at any time *t*, can be decomposed as follows:


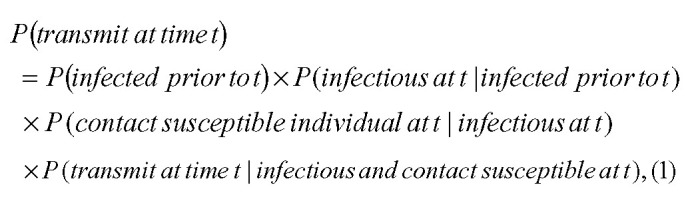



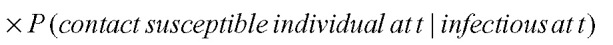



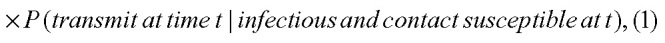


and so:


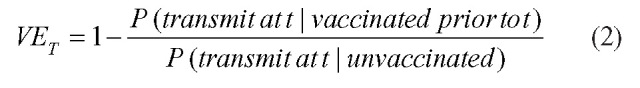


Under the assumptions that: the incidence rate in each arm of the trial is constant across time (Assumption 1); and vaccination status does not affect the probability of having contact with a susceptible individual given that an individual is infectious (Assumption 2), the vaccine efficacy against transmission is given by:


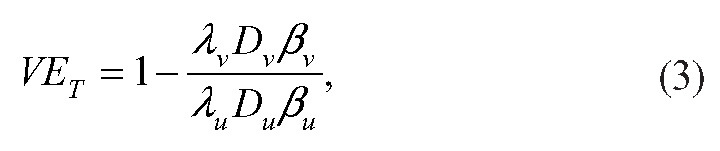


where 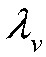 is the incidence rate of infection among vaccinated individuals, 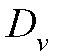 is the average duration of infectiousness for infected vaccinated individuals, and 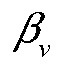 is the average per-contact transmission probability from an infected vaccinated individual; these quantities with subscript *u* refer to the quantities among unvaccinated individuals.

Mathematically, this can be rewritten as:


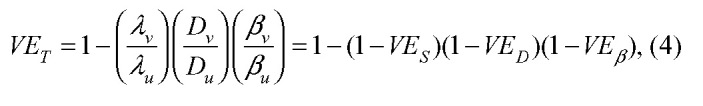


where 
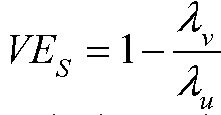
 is the vaccine efficacy against susceptibility or viral acquisition, 
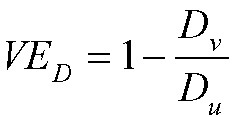
 is the vaccine efficacy against duration of infectiousness, and 
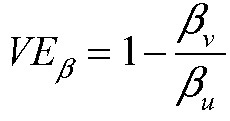
 is the vaccine efficacy against per-contact infectiousness, defined as the reduction in the probability of transmission per contact._7,13_ Halloran et al. use 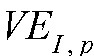
where we use 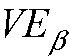; we avoid that notation to avoid confusion with vaccine efficacy against progression._7,13_

Under the assumption that an individual is infectious only if they have detectable viral load (Assumption 3), then we can write:


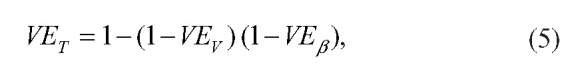


where 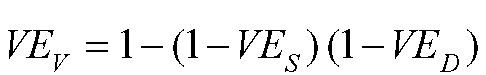 is the vaccine efficacy against prevalent detectable infection, per Lipsitch and Kahn,^17^ and 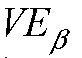 is again the vaccine efficacy against per-contact infectiousness.

## ESTIMATOR USING RANDOM SAMPLES FROM VACCINE RCT

By equation 5, to estimate 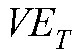, it suffices to estimate 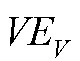 and 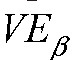. In a vaccine RCT, 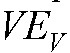 can be estimated using the odds ratio of prevalent detectable infection at time *t* among vaccinated individuals vs. unvaccinated individuals.^16–18^

If a random sample of participants have virologic tests conducted at some time *t* after enrollment in an RCT, then the measured viral loads from these test results can be used to estimate the distribution of true viral loads among the vaccinated and the unvaccinated individuals. Note that a nonrandom sample—e.g., tests conducted upon onset of symptoms or upon contact tracing identification, as in Brown et al.^8^—would not provide exactly the distribution of viral loads across the course of infection necessary for this approach.^21^ As above, we assume that Assumptions 1–3 hold. If we also assume that the per-contact infectiousness of an individual among vaccinated individuals is some known function 
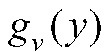
 of the measured viral load *y*, and the per-contact infectiousness of an individual among unvaccinated individuals is some known function 
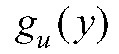
 (Assumption 4), then:


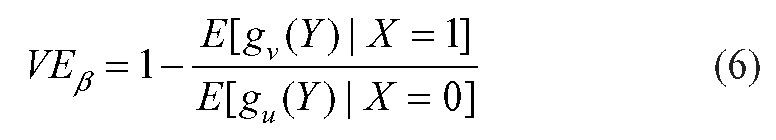


where *Y* is the measured viral load of a randomly chosen infectious individual and *X* is the vaccination status of that individual, where 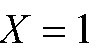 denotes a vaccinated individual and 
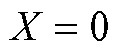
 an unvaccinated individual. Note that if vaccination only affects infectiousness through its effect on viral load (that is, viral load fully mediates the vaccine efficacy on infectiousness), then 
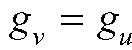
. These assumptions are described in more detail below.

Define the estimator:


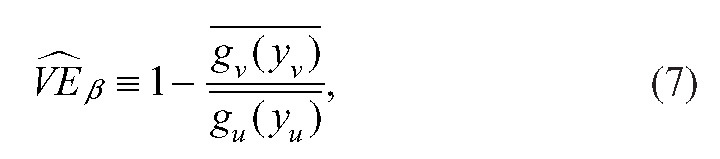


where 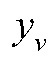 is the measured viral load of an individual in the vaccinated arm of the trial and 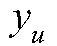 the measured viral load of an individual in the unvaccinated arm of the trial, and the overbars represent averages across all sampled individuals in the respective arms of the trial. Then if the infectiousness functions (*g*_*v*_ and *g*_*u*_) are correctly specified with respect to the measured viral load in the sample, 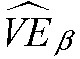 is a consistent estimator of 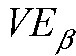.

If a measured covariate (e.g., symptom status) modifies the effect of viral load on per-contact infectiousness, this can be incorporated by specifying infectiousness functions for each level of the covariate. For example, let *S* denote the symptom status of an individual (equal to 1 if symptomatic and 0 if not). Then, if the infectiousness function among symptomatic individuals, whether vaccinated or not, is given by *g*_*1*_ and the infectiousness function among individuals not currently symptomatic is given by *g*_*0*_, then:


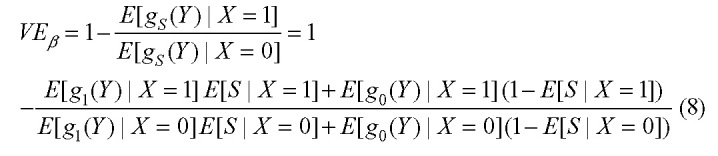


This can be estimated by:


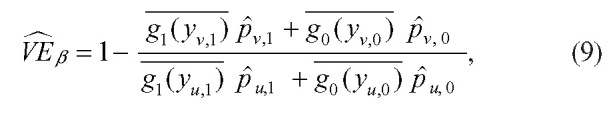


where 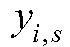 represents the measured viral load of an individual in arm *i* with symptom status *s*, the overbars represent averages over the sampled individuals in that arm with that symptom status, and 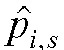
is the proportion of individuals in arm *i* who have symptom status *s*, for 
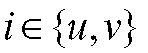
 and 
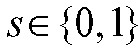
.

If a study is conducted with randomly sampled virologic tests conducted at a time point *t*, the conditions for a consistent estimator 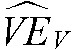 are met,^15^ and the conditions described above for a consistent estimator 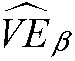 are met, then these can be combined to get a consistent estimator of 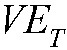:


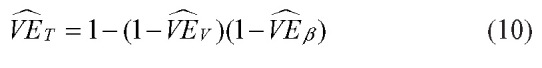


## ASSUMPTIONS FOR CONSISTENCY

As derived in the previous section, the consistency of 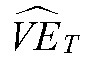 as an estimator of *VE*_*T*_ relies on four key assumptions. In this section, we describe these assumptions, the consequences of violations of the assumptions, examples of potential violations, and solutions to address these violations. The Table provides a summary of these assumptions.

### Assumption 1: Within Each Arm of the Trial, the Incidence Rate Among Participants Is Constant Throughout Time

Under this assumption, there is a baseline incidence rate within each arm of the vaccine trial, which differs between the arms only due to the vaccine efficacy against susceptibility. If this assumption is violated, then the distribution of viral loads in each arm, *Y*_*v*_ and *Y*_*u*_, measured by cross-sectional testing at a single point in time are not representative of the distribution of viral loads across the course of infection.^21^

This assumption is violated if the epidemic is growing or waning in the communities in which the trial is conducted. For example, in a growing epidemic, the average time since infection for cases ascertained in a cross-sectional sample is lower than in a flat epidemic.^21–23^ If the vaccine efficacy on viral load mostly affects the later stage of infection—for example, by allowing faster viral clearance, as has been suggested for the Delta variant^20^—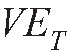 will be underestimated in this sample. In a waning epidemic, more individuals will be in the later stage of infection, and so 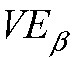 will be overestimated in this sample. If, on the other hand, the vaccine efficacy on viral load mostly affects the earlier stage of infection (e.g., by reducing the peak viral load), the reverse will happen: 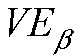 will be underestimated in a growing epidemic and overestimated in a waning epidemic. Data on epidemic trajectory could be used to adjust for this bias. Follmann and Fay consider this bias for some viral load functions and vaccine efficacy parameters and find that the bias is less than 10% for the scenarios considered.^18^

Without adjusting for this bias, the estimate represents the instantaneous vaccine effectiveness on transmission at the time of sampling. That is, the estimand is a function of the epidemic conditions in the community where the trial was conducted. This quantity might be useful in generalizability to communities at a similar stage of their epidemic trajectory.

This assumption is complicated if there are multiple variants of the pathogen with different incidence rates in the study population. In particular, even if the overall incidence of infection is constant, the assumptions can be violated if one variant is increasing while another is decreasing. If the vaccine has different efficacy levels against the variants, as has been suggested for several SARS-CoV-2 vaccines,^8,24–26^ the observed vaccine efficacy represents a weighted average of these efficacies. However, if the variants have different viral load patterns in infection, as has been suggested for multiple SARS-CoV-2 variants,^20,27–29^ the observed efficacy would then be a weighted average of the specific point-in-time efficacies against each variant at its current growth or decline rate of incidence, which is unlikely to be generalizable to any other population.

### Assumption 2: Vaccination Status Does Not Affect the Probability of Contact With a Susceptible Individual at Any Point in Time

Under this assumption, individuals in both arms have the same distribution of contact rates. If this assumption is violated, then the probability of transmission is no longer proportional to the product of the probability of infection, the mean duration of infectiousness, and the mean per-contact infectiousness (i.e., equations 3 and 4 no longer hold). Correcting for violations of this assumption is challenging as the effects depend on changes in behavior as a result of vaccination.

For example, this violation may occur, even in an RCT with concealed allocation, if vaccination reduces the probability of symptoms given infection (vaccine efficacy for progression to symptoms > 0) and symptomatic individuals are less likely to contact others than asymptomatic individuals. In this case, data on symptom status of cases could be used to adjust for this effect. Additionally, risk compensation of vaccinated individuals—either during nonrandomized vaccine rollout, in an open-label trial, or in a trial with concealed allocation where self-unblinding occurred intentionally^30^ or through strong reactogenicity in the vaccine arm^2,4^—may affect behavior and contact patterns, violating this assumption.^31^

This assumption is especially important and challenging to ensure in an observational study. During vaccine rollout, vaccinated individuals may have different baseline behaviors from those who are not vaccinated, biasing observational estimates of vaccine effectiveness. Vaccinated individuals may be more likely to interact with other vaccinated individuals, either intentionally or by assortative mixing due to the nature of social networks (e.g., in a cluster randomized trial or during geographically phased vaccination rollout), as has been shown for other vaccines,^32^ or they may engage in risk compensation and relax other precautionary measures.^31^ Contact surveys or proximity data from mobile phones could be used to understand the magnitude of this effect,^33,34^ or it could be estimated by the risk of infection after vaccination but before its effects occur.^35^ These quantitative estimates can then be incorporated into the vaccine efficacy estimate as a component of the probability of contacting a susceptible individual described earlier. These potential changes in behavior and contacts may differentiate the vaccine efficacy measured in an individual RCT from the vaccine effectiveness during a wide rollout or cluster randomized trial; the validity of observational measures of this quantity should thus be considered with caution.

**FIGURE 1.**
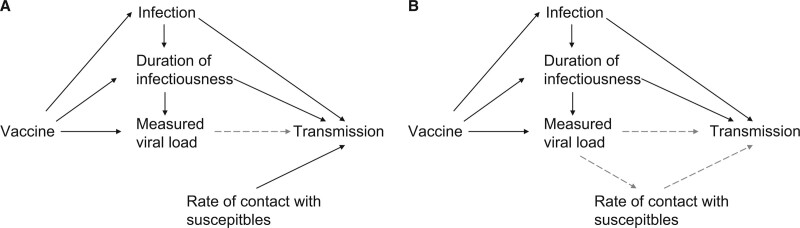
Directed acyclic graph for assumption 2. Vaccination can affect transmission through its effect on infection, its effect on duration of infectiousness, and its effect on the measured viral load, a proxy for its effect on infectiousness at any point in time through the infectiousness function *g* (dashed arrow). The rate of contact with susceptibles, however, is not affected by the vaccine (A). If the rate of contact with susceptibles is affected by the viral load of the infected individual (e.g., through changing contact patterns because of symptoms), the infectiousness function *g* (dashed arrows) must account for this relationship between measured viral load and transmission probability (B).

This assumption can be slightly relaxed if the infectiousness function relating measured viral load and per-contact infectiousness actually measures infectiousness accounting for changes in contact patterns that are fully mediated by changes in measured viral load. For example, suppose that higher viral loads are associated with more severe illness, limiting contacts per time infected. If the function *g* was estimated from a study that estimated secondary attack rate as a function of viral load,^36^ this change in contact pattern, mediated by the viral load, would be incorporated into *g* and thus into the vaccine efficacy estimate here. Interpretation of 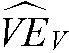 should consider the appropriate interpretation of *g*. Figure 1 shows directed acyclic graphs for both versions of this assumption.

### Assumption 3: Individuals Without Detectable Viral Load Are Not Infectious

This assumption implies a perfectly sensitive test for infectiousness. Under a violation of this assumption, for example because of low sensitivity, 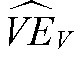 will be biased.^17^ Adjustments for test characteristics could alleviate the inconsistency in 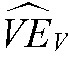. However, the estimate of 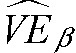 must also be adjusted to account for the infectiousness of undetected infections. This adjustment is less straightforward, as it requires an estimate for the infectiousness of individuals who are infectious but have an undetectable viral load. Sensitivity analysis could be conducted with different assumptions about the infectiousness of these individuals, for example, by assuming they are no more infectious than the average individual with a detectable viral load.

Note that this does not make any assumption about the specificity of the test used. Imperfect specificity can cause bias in 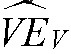, but if this is properly accounted for in the infectiousness function, as described under the next assumption, the estimator 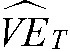 will remain consistent.

### Assumption 4: Vaccination Affects Per-contact Infectiousness Only Through the Measured Viral Load (and Other Measured Covariates); This Relationship Follows a Specified Function or Functions

Correct specification of the relationship between the measured viral load and infectiousness is crucial to the estimation of 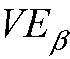. In other words, the measured viral load acts as a surrogate endpoint for transmission.^37^ Although viral load has been suggested as a proxy for infectiousness of SARS-CoV-2,^8,36,38–41^ direct evidence that it meets the formal operational criteria for a surrogate endpoint is limited. There are several reasons this assumption may be violated.

First, this assumption would be violated if vaccination reduces the likelihood of being symptomatic at each viral load level and symptomatic patients have higher infectiousness per contact. Data on symptom status could be used to adjust for this and perhaps separate models of the relationship could be used based on symptom status, as described above. This requires, however, the specification of more infectiousness functions.

Second, this assumption would be violated if measured viral load is not an accurate summary of the true viral load and the infectiousness function does not capture this measurement error. This could occur, for example, if the relationship between viral load and infectiousness is estimated using a specific testing platform and the viral loads are measured with a different platform that has different measurement error properties. It could also occur if the relationship is estimated from viral load measurements at a single time point during infection (e.g., symptom onset), as that will be an imperfect representation of the full trajectory of viral loads during infection. As an example, studies culturing viral samples have come to different conclusions about which threshold of Ct value corresponds with infectious virus, possibly due to different sampling methods, testing platforms, or populations.^42,43^ Ideally, investigators will validate the infectiousness relationship using the same laboratory conditions as in the trial itself.

More generally, the relationship between infectiousness and viral load may be misspecified or unknown, especially for vaccinated individuals. This could occur because the relationship is specified on the wrong scale (e.g., proportional to the log viral load or Ct value rather than to the linear viral load, or a threshold effect), because of measurement error (e.g., the discrete nature of Ct values), or uncertainty in the relationship. It might still be possible, however, to get a range of reasonable estimates for 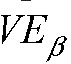 by conducting sensitivity analyses with a variety of potential infectiousness functions. 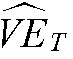 can then be bounded by calculating its value with extreme possibilities of these 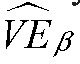 values. For example, infectiousness proportional to the viral load and infectiousness constant above a relatively low viral load threshold may be reasonable bounds for most pathogens. These bounds would, for example, encompass a true infectiousness function that is proportional to the log of the viral load.

Violations can occur specifically if vaccination itself affects the relationship between viral load and infectiousness in a way that is not mediated by symptom status or other measured covariates. Often, the only available data to estimate the infectiousness function will be from studies conducted before vaccination, so there may be no ability to account for this difference. For example, Regev-Yochay et al. find that the PCR-positive individuals with a Ct value below 30 were less likely to test positive for antigen if vaccinated than if unvaccinated.^38^ If, as has been suggested by viral culture studies,^44^ antigen tests are a more accurate reflection of infectiousness than PCR positivity with Ct values below 30, then this would indicate that vaccines can reduce transmissibility through a mechanism not mediated fully by PCR viral load. If, for any viral load level, a vaccinated individual is no more infectious than an unvaccinated individual, assuming 
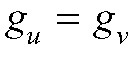
 would yield a lower bound for 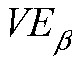.

**FIGURE 2.**
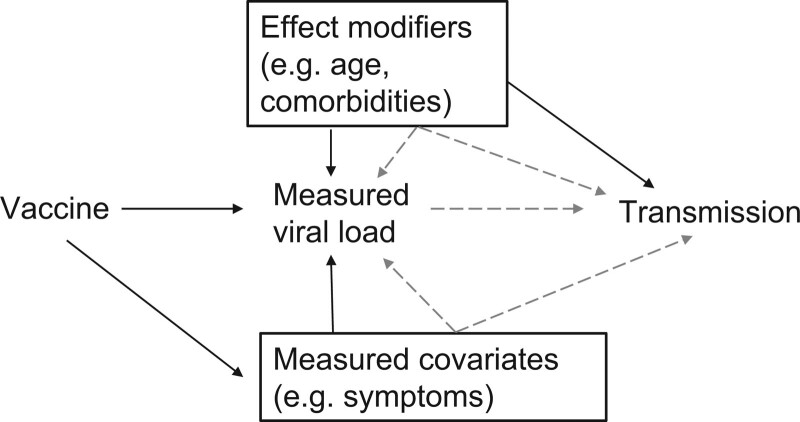
Directed acyclic graph for assumption 4. Vaccination can affect transmission only through the measured viral load and measured covariates (e.g., symptom status). Any effect modifiers of the relationship between the measured viral load at any point in time and transmission (e.g., age, comorbidities) are measured and adjusted for through the infectiousness function *g* (dashed arrows).

Figure 2 shows a directed acyclic graph displaying these assumptions. In practice, this element of the analysis will rely on epidemiologic studies conducted to estimate this relationship,^36,41,45^ and laboratory analyses that improve biologic understanding of the transmission process.^39,40^ For SARS-CoV-2, these studies suggest a range of possible infectiousness functions: categorical relationships between Ct values or viral loads and infectiousness,^36,45^ logistic models,^39,41^ and more complicated relationships.^40^ However, these studies are limited in their application to this method because they generally measure viral load at the time of contact tracing identification or onset of symptoms, rather than at a randomly sampled time in the duration of infection. Until an epidemiologic study with randomly sampled viral loads is conducted, uncertainty will remain in the measurement of this relationship. Investigators can account for the uncertainty inherent in this relationship via. the sensitivity analyses (e.g., analyzing results using different infectiousness functions) or by formal Bayesian inference that incorporates prior distributions on the infectiousness function or its parameters. How different infectiousness functions will affect the final estimates will depend not only on the structure of those functions but also how the vaccine affects viral loads, and so general bounds on functions are not particularly useful.

## DISCUSSION

Understanding the efficacy of vaccines and how they will affect the trajectory of the SARS-CoV-2 pandemic requires a wide array of studies, both randomized and observational. To get the most information out of large-scale RCTs, these trials should incorporate virologic testing of a random sample of participants, as suggested previously.^17,18^ This allows estimation of vaccine efficacy against prevalent infection, rather than just symptomatic infection. We have shown that, under additional assumptions, using the quantitative information from such random testing additionally allows for the estimation of vaccine efficacy against transmission, a crucial parameter for understanding how widespread vaccination will affect circulating virus.

This estimation requires four key assumptions. First, the incidence rate among participants must be constant throughout time within each arm of the trial. Second, vaccination status must not affect the probability of contact with a susceptible individual during the study. Third, individuals without a detectable viral load are assumed to be uninfectious. And fourth, vaccination must affect infectiousness only through a known function of the measured viral load and, potentially, other measured covariates.

Meeting the assumptions for this estimation requires an understanding of the role of viral load in the transmission process. This increased understanding of viral load and proxies such as the Ct value measured by RT-qPCR tests can contribute to better understanding of the pandemic in several ways, including monitoring population incidence,^21^ assessing the role of variants in outbreaks,^8,27–29^ assessing test performance,^46^ and clinical management.^47^ Although some studies have assessed the association between measured viral load and infectiousness,^36,41,45^ more such work is needed to improve this aspect of the estimation.

For SARS-CoV-2, existing studies can be used to assess these assumptions, but in any specific setting they are likely unprovable. For assumption 1, running the trial during a time period where incidence is flat is key; if this is not possible, investigators should acknowledge the lack of generalizability of the observed Ct values^21^ and the uncertainty that brings, as shown elsewhere.^18^ In the closed-label individually randomized trial setting, assumption 2 is similar to a general assumption of exchangeability in RCTs. The generalizability to a wide vaccine rollout, however, depends on behavioral patterns and an assumption that risk compensation will not occur. Assumption 3 relies on the sensitivity of the test, which has been well-established for SARS-CoV-2.^46^ Assumption 4, the most challenging assumption, can be estimated from a variety of existing studies,^36,39–41,45^ which can be used to provide a range of estimates for the vaccine efficacy. If feasible, a parallel study to the main trial specifically designed to determine the infectiousness function for the test instrument used in the trial on the viral population circulating at that time, for example, by culturing or epidemiologic tracing, would be beneficial and allow more precise specification by investigators.

This method could be used to compare the efficacy of two different vaccines or vaccine regimens and in observational studies, in which control of confounding and avoidance of selection biases will present challenges similar to, but slightly more extensive than, the ones in standard vaccine effectiveness studies.^48^ In addition to the four main assumptions, such an analysis would require exchangeability assumptions: that there is no unmeasured confounding between vaccination status and any of the following: the probability of infection, duration of infection, measured viral load, and the relationship between viral load and infectiousness. Moreover, for a retrospective study, selection criteria would need to not exclude individuals because of any factor—for example, hospitalization or death—causally related to vaccination and measured viral load^49^ and would need random sampling of viral loads across the course of infections.

Limiting the spread of SARS-CoV-2 variants, ending the SARS-CoV-2 pandemic, and mitigating future pandemics will require the best available evidence, as quickly as possible, on the full set of measures of vaccine efficacy. We have described a method to gain a richer picture of vaccine efficacy on transmission with the optimal use of quantitative data from virologic testing on a sample of trial participants.

**TABLE.** Summary of Assumptions for Consistent Estimation

	Assumption	Consequences of Violation	Example of Violation	Possible Solution(s)
1	There is a constant incidence rate ( 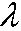 ) across time within each treatment arm (but it may differ across treatment arms due to vaccine effect, 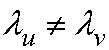 )	The prevalence odds will no longer be proportional to the product of incidence and mean duration. The transmission probability will no longer be proportional to the product of the mean duration time and the mean infectiousness in the sample. Equations 3–5 fail.	(1) If the epidemic is growing or waning in the communities in which the trial is conducted. (2) If 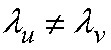 then prevalence odds in vaccinated will reflect a mix of incidence before and after the vaccine took effect.	Use outside information to adjust distributions for epidemic trajectory. Estimates at different time points in an outbreak could be useful for other communities with similar epidemics.
2	Vaccination status does not affect probability of contact with susceptible individual at any time point	Probability of transmission is no longer proportional to the product of the probability of infection, the mean duration of infectiousness, and the mean infectiousness given contact. Equations 3–5 fail.	(1) If vaccination reduces the probability of symptoms given infection and symptomatic patients are less likely to contact other individuals. (2) If vaccinated individuals increase risky behavior in a rollout or if there is unblinding in a trial. (3) In a rollout or cluster randomized trial, if vaccinated individuals are more likely to come into contact with other vaccinated individuals rather than unvaccinated susceptible individuals.	If the effect is through an effect on symptoms or another measured covariate, the infectiousness function can be adjusted. This estimate might distinguish the vaccine efficacy measured in an individual randomized controlled trial from the vaccine effectiveness seen in a cluster randomized controlled trial or a real-world rollout.
3	Undetectable individuals are uninfectious	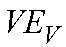 no longer measures vaccine efficacy against being infectious. Equation 5 fails.	Low test sensitivity.	Might be able to adjust if sensitivity is known, but need to account for relative infectiousness of undetectable individuals.
4	Vaccination affects per-contact infectiousness only through the measured viral load (and other measured covariates), via. a known function or functions	Comparison of viral loads no longer estimates 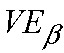 . Equation 6 fails.	(1) If vaccination reduces the likelihood of being symptomatic at each viral load level and symptomatic patients have higher infectiousness per contact. (2) If viral load measured at a point in time is an inaccurate summary of mean viral load and the infectiousness function does not correctly account for this mismeasurement. (3) If the relationship between viral load and infectiousness is unknown or mismeasured, or the effect of vaccination on this relationship is unknown.	If it affects a covariate (e.g., symptom status) that is measured in the sample, then the infectiousness functions can be specified conditional on the covariate. Can use hypothesized relationships to get bounds on 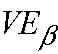 . Can conduct sensitivity analyses with other possible relationships.
